# Three-dimensional pseudocontinuous arterial spin labeling perfusion imaging shows cerebral blood flow perfusion decline in attention-deficit/hyperactivity disorder children

**DOI:** 10.3389/fpsyt.2023.1064647

**Published:** 2023-01-18

**Authors:** Shilong Tang, Xianfan Liu, Lisha Nie, Fangfang Qian, Wushang Chen, Ling He

**Affiliations:** ^1^Department of Radiology, Children’s Hospital of Chongqing Medical University, National Clinical Research Center for Child Health and Disorders, Ministry of Education Key Laboratory of Child Development and Disorders, Chongqing Key Laboratory of Pediatrics, Chongqing, China; ^2^GE Healthcare, MR Research China, Beijing, China

**Keywords:** children, attention-deficit/hyperactivity disorder, magnetic resonance imaging, brain, 3D-pcASL

## Abstract

**Purpose:**

To investigate the feasibility of three-dimensional pseudocontinuous arterial spin labeling (3D-pcASL) perfusion imaging in the brain of children with Attention-deficit/hyperactivity disorder (ADHD).

**Methods:**

A total of 78 ADHD children aged 5–13 years were prospectively selected as the study group, and 89 healthy children matched in age and sex were selected as the control group. All children underwent MRI conventional sequence, 3D-pcASL, and 3D-T1 sequence scans. The brain gray and white matter volume and cerebral blood flow (CBF) perfusion values were obtained by software post-processing, and were compared and analyzed in the two groups to find out their characteristics in the brain of ADHD children.

**Results:**

The total brain volume and total CBF values were lower in ADHD children than in healthy children (*P* < 0.05); the gray and white matter volumes in the frontal lobe, temporal lobe, hippocampus, caudate nucleus, putamen, globus pallidus and other brain regions were lower in ADHD children than in healthy children (*P* < 0.05); the gray matter CBF values in the frontal lobe, temporal lobe, hippocampus, caudate nucleus, putamen, globus pallidus and other brain regions were lower in ADHD children than in healthy children (*P* < 0.05); the differences between the white matter CBF values of white matter in the said brain regions of ADHD children and healthy children were not statistically significant (*P* > 0.05); and the CBF values in frontal lobe and caudate nuclei could distinguish ADHD children (AUC > 0.05, *P* < 0.05).

**Conclusion:**

The 3D-pcASL technique showed reduced cerebral perfusion in some brain regions of ADHD children.

## Background

Attention-deficit/hyperactivity disorder (ADHD) is one of the most common mental diseases in childhood. Patients present with three core symptoms, including inattention, hyperactivity, and impulsivity, which are not age-appropriate, and are often accompanied by emotional problems, learning problems, and conduct disorders. It seriously affects children’s academic and emotional development (1–6). ADHD is multifactorial and is associated with neurodevelopment, genetics, and some other factors that are not well understood, such as the abnormal development of brain regions such as frontal lobes and basal ganglia in ADHD children, but it is not known whether the blood perfusion in the abnormal developmental brain regions is abnormal (7–10). Some studies showed that abnormal brain function may result in abnormal brain structure, and abnormal brain function usually precedes abnormal brain structure (11–13). Therefore, it is helpful for early diagnosis of ADHD by knowing whether abnormal cerebral blood perfusion exists in ADHD children.

In previous studies, some researchers have used single photon emission computed tomography (SPECT) to understand patients’ cerebral blood flow (CBF) perfusion, in which radioactive isotopes are used as tracers to obtain blood flow values in various brain regions. As isotopes are radioactive and hazardous to humans, how to adopt a technique that does not affect patients to obtain CBF is one of the research hotspots for medical researchers (14–18).

Three-dimensional pseudocontinuous arterial spin labeling (3D-pcASL) CBF perfusion imaging is a technique that continuously labels the arterial blood flowing into the brain, and performs rapid 3D imaging of the whole brain and measures changes in blood flow throughout the brain after the labeled blood has flowed into the brain tissue. This technique is non-invasive and can repeatedly image and evaluate the function of the whole brain without contrast injection, and has been widely used in clinical practice (19–23). Currently, many studies have been reported on the application of 3D-pcASL technique to brain examination in adults, while fewer studies have been reported on the application to brain examination in children, especially in ADHD children (24–27).

We obtained blood perfusion values in various brain regions of children by 3D-pcASL CBF perfusion imaging, and compared and analyzed the same in ADHD children and healthy children, to find out the difference of CBF values between ADHD children and healthy children, so that we can accurately identify the CBF characteristics of ADHD children as early as possible, and then they can be timely diagnosed and properly treated.

## Data and methods

The study was approved by the Ethics Committee of the Children’s Hospital of Chongqing Medical University (NO. 2019-221), and the families of children under study signed an informed consent form before the examination.

### General data

Study group: 86 ADHD children from May 2019 to April 2022 were prospectively selected and 78 were included in the study; Control group: 103 healthy children from June 2019 to April 2022 were prospectively selected and 89 were included in the study; children not included in the study were those who failed the examination or had abnormal lesions in the brain, and all children underwent MRI examinations while awake (Table 1).

**TABLE 1:** Patient information.

Information				
Group	Male to female ratio	Age (years)	Weight (kg)	BMI
ADHD children	41:37	8.57 ± 2.38	29.36 ± 4.56	17.56 ± 1.89
Healthy children	46:43	8.67 ± 2.56	28.89 ± 3.82	17.68 ± 2.23
X^2^/*T*-value	0.079	−0.576	0.162	−0.006
*P*-value	0.763	0.538	0.875	0.982

Inclusion criteria for healthy children: those children with right-handedness, no functional neurological disorders, no concomitant diseases of other organs, no other diseases that may affect brain function and structure, and no abnormalities in routine brain MRI examinations, and all children had body mass indexes within normal limits.

Inclusion criteria for ADHD children: those child patients with right-handedness, meeting the Diagnostic and Statistical Manual of Mental Disorders (DSM-V) criteria for ADHD, no functional neurological disorders, no concomitant disorders of other organs, no other disorders that may affect brain function and structure, no history of previous medication, and no abnormalities on routine brain MRI.

All ADHD children were diagnosed by senior doctors in the Department of Psychology, Children’s Hospital of Chongqing Medical University. The ADHD children included in the study met the DSM-V criteria for ADHD (Edition 5). All ADHD children included in the study were diagnosed for the first time, and no psychotropic drugs were used before MRI examination.

### Devices and methods

A GE discovery MR750 3.0T MRI scanner with an 8-channel combined head and neck coil was used. All children were scanned with cerebral MRI conventional sequence, 3D-T1 sequence and 3D-pcASL sequence, where the conventional sequence includes T1 FLAIR, T2 FLAIR, and T2 WI sequences in horizontal axis. T1 FLAIR imaging parameters were FOV 24 cm × 24 cm, TR 1750 ms, TE 24 ms, NEX 2 times, IT 720 ms, Flip Angle 111, layer 16, thickness 6 mm, spacing 1 mm; T2 FLAIR imaging parameters were FOV 24 cm × 24 cm, TR 9000 ms, TE 120 ms, NEX 1 times, Flip Angle 160, layer 16, thickness 6 mm, spacing 1 mm; T2 WI imaging parameters were FOV 24 cm × 24 cm, TR 1869 ms, TE 110 ms, NEX 1.5 times, layer 16, thickness 6 mm, spacing 1 mm; 3D-pcASL imaging parameters were TR 4628 ms, PLD 1525 ms, FOV 25 cm × 25 cm, TE 10.4 ms, NEX 3 times, layer thickness 4.2 mm, number of acquired layers 32, scan time 4 min 29 s; 3D-T1 imaging parameters were TR 450 ms, FOV 25 cm × 25 cm, TE 3.1 ms, NEX 1 time, layer thickness 1 mm, number of acquired layers 152, acquisition time 3 min 43 s.

**FIGURE 1:**
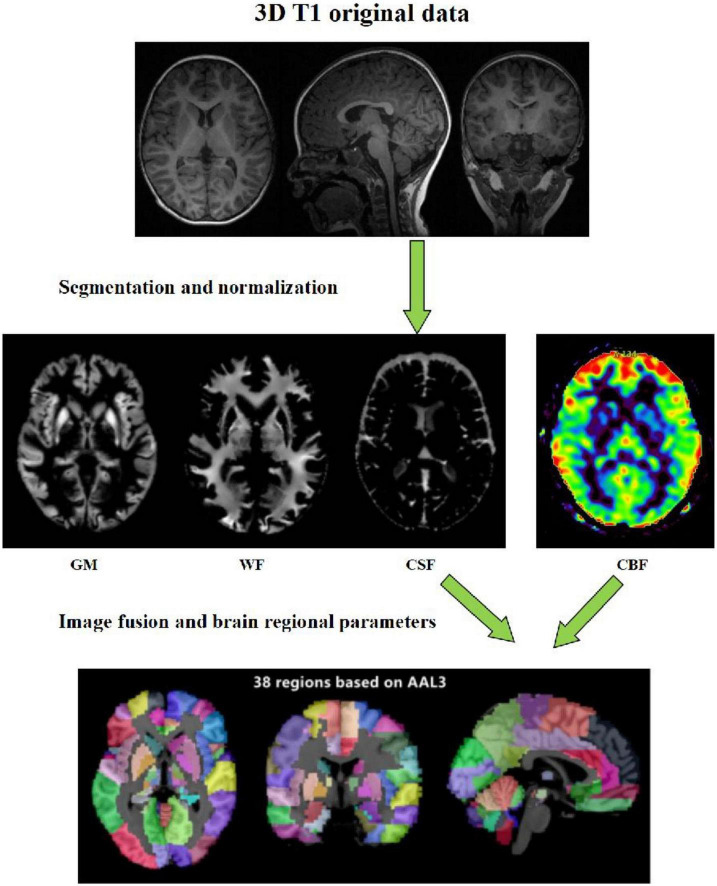
Schematic diagram of the image processing and parameter extraction.

Data analysis: The raw data of 3D-pcASL sequences were imported into Functools software using GE ADW 4.6 workstation to obtain the CBF quantitative maps. In order to calculate the values of volumes, CBF and other quantitative parameters in different brain regions, we used the voxel-based morphometry (VBM) method. On the platform of MATLAB 2018a, we used SPM12 software to register the 3D-T1 sequence structure diagram with CBF quantitative maps, and used CAT12 toolkit to segment the registered CBF structure quantitative maps in SPM12 software, and finally extracted the parameter values of volume and CBF in each brain region (Figure 1).

### Statistical analysis

SPSS 25.0 statistical software was used, and the measurement data were expressed as *x̄* ± s. A chi-square test was used to compare the sex of children in the two groups, a two-independent samples *T*-test was used to compare the age, weight, and body mass index of children in the two groups, and the same was used to compare the CBF values of 3D-pcASL perfusion in the same brain regions of children in the two groups (*P* < 0.05 was considered a statistically significant difference); a two-independent samples *T*-test was used to compare the volumes of the same brain regions between the two groups of children (*P* < 0.05 was considered a statistically significant difference).

A receiver operating curve (ROC) analysis was used to evaluate the diagnostic value of CBF values for the diagnosis of ADHD in children. AUC greater than 0.5 and statistically significant was considered to have diagnostic value, and the closer the value was to 1 the higher the diagnostic value.

## Results

### Comparative results of CBF values in the same brain regions

**FIGURE 2:**
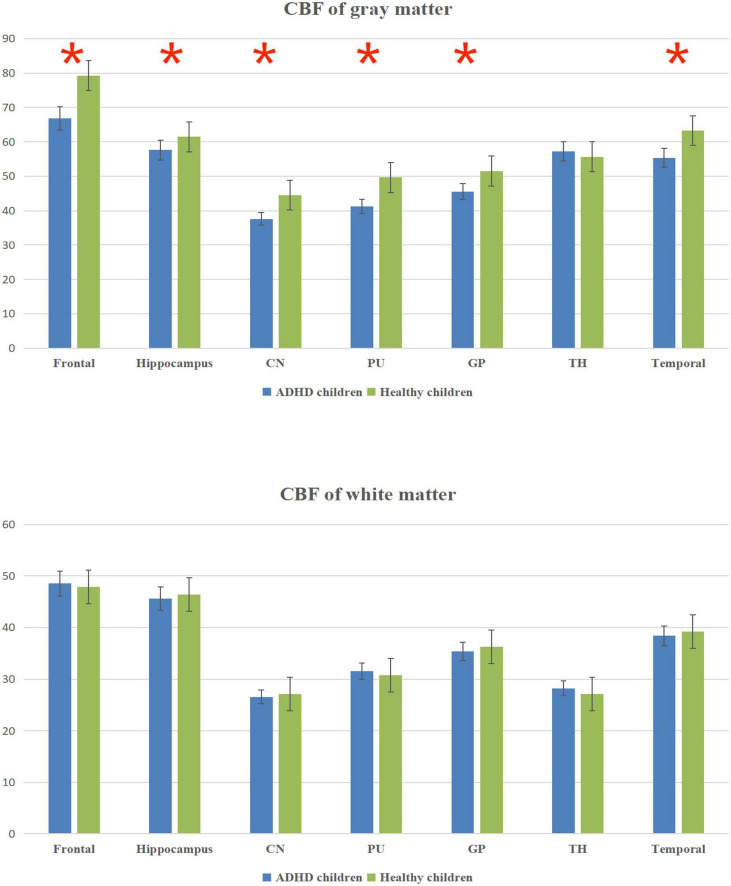
Cerebral blood flow (CBF) column chart of gray matter and white matter. *Statistical significance at the level of 0.05.

The total CBF was lower in ADHD children than in healthy children (*p* < 0.05); the gray matter CBF values in the frontal lobe, temporal lobe, hippocampus, caudate nucleus, putamen, globus pallidus and other brain regions were lower in ADHD children than in healthy children (*P* < 0.05); the differences between the white matter CBF values of white matter in the said brain regions of ADHD children and healthy children were not statistically significant (*P* > 0.05); (Figure 2 and Table 2).

**TABLE 2:** Comparison of the CBF value between the ADHD and healthy children groups (mL/100 g⋅min).

Brain regions							
Group	Frontal	Hippocampus	CN	PU	GP	TH	Temporal
**Gray matter**							
ADHD children	66.81 ± 5.27	57.59 ± 7.13	37.63 ± 4.87	41.26 ± 5.65	45.63 ± 7.69	57.23 ± 6.57	55.34 ± 6.89
Healthy children	79.27 ± 6.86	61.43 ± 5.68	44.52 ± 5.43	49.65 ± 7.42	51.52 ± 12.26	55.62 ± 7.69	63.28 ± 6.24
*T*-value	−2.762	−3.237	−3.166	−0.617	−3.238	0.467	−2.215
*P*-value	0.003	0.002	0.002	0.004	0.001	0.635	0.039
**White matter**							
ADHD children	48.52 ± 5.35	45.59 ± 6.22	26.54 ± 5.15	31.52 ± 5.71	35.34 ± 4.83	28.23 ± 6.32	38.38 ± 7.25
Healthy children	47.86 ± 6.29	46.42 ± 5.89	27.14 ± 5.14	30.78 ± 4.89	36.29 ± 6.07	27.15 ± 7.65	39.23 ± 6.57
*T*-value	2.347	−3.218	−0.987	0.934	−2.156	1.322	−2.343
*P*-value	0.073	0.062	0.325	0.069	0.171	0.239	0.242
**Whole brain**							
ADHD children	56.33 ± 6.78						
Healthy children	63.24 ± 6.61						
*T*-value	−1.369						
*P*-value	<0.001						

### Comparative results of volumes in the same brain regions

**FIGURE 3:**
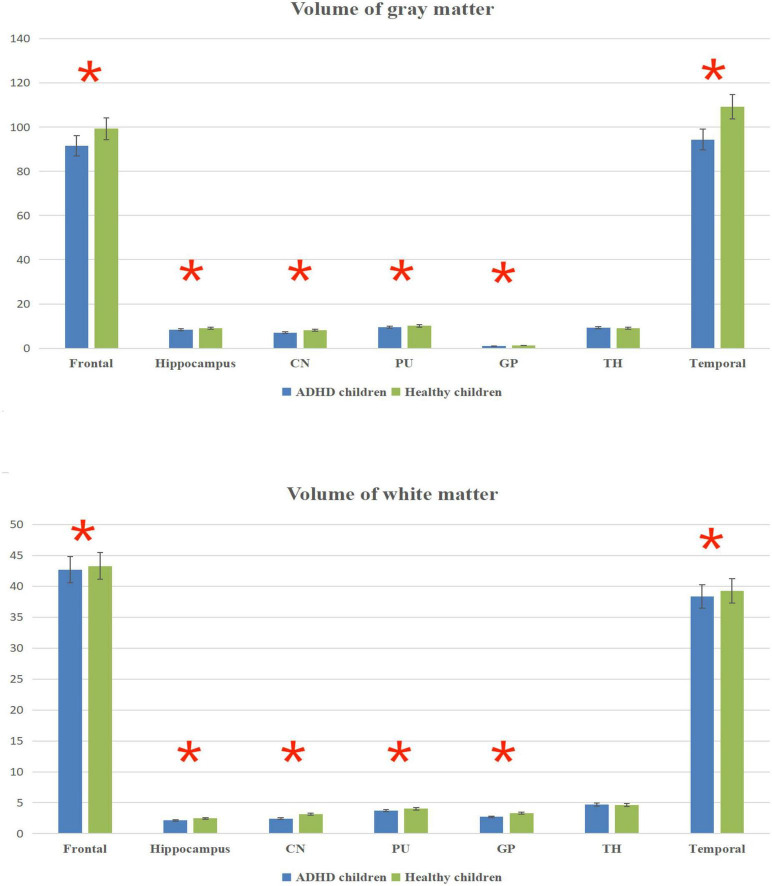
Volume column chart of gray matter and white matter. *Statistical significance at the level of 0.05.

The total brain volume was lower in ADHD children than in healthy children (*P* < 0.05); the gray and white matter volumes in the frontal lobe, temporal lobe, hippocampus, caudate nucleus, putamen, globus pallidus and other brain regions were lower in ADHD children than in healthy children (*P* < 0.05) (Figure 3 and Table 3).

**TABLE 3:** Volume values of brain regions in children [$\bar{x}$ ± s, volume (mm^3^)].

Brain regions							
Group	Frontal	Hippocampus	CN	PU	GP	TH	Temporal
**Gray matter**							
ADHD children	91.56 ± 11.69	8.36 ± 1.25	7.12 ± 1.15	9.52 ± 1.71	0.98 ± 0.33	9.23 ± 1.98	94.34 ± 7.25
Healthy children	99.24 ± 11.68	9.12 ± 0.89	8.14 ± 1.23	10.18 ± 1.89	1.29 ± 0.47	9.15 ± 1.43	109.23 ± 9.13
*T*-value	−1.782	−1.264	−0.975	−0.932	−2.655	0.378	−1.357
*P*-value	<0.001	<0.001	<0.001	<0.001	<0.001	0.065	<0.001
**White matter**							
ADHD children	42.64 ± 5.68	2.15 ± 0.76	2.43 ± 1.15	3.73 ± 1.71	2.73 ± 1.83	4.72 ± 0.98	38.34 ± 7.26
Healthy children	43.29 ± 5.61	2.46 ± 0.83	3.14 ± 1.13	4.05 ± 1.89	3.29 ± 1.97	4.65 ± 0.83	39.23 ± 6.14
*T*-value	−1.412	−1.579	−1.616	−0.976	−2.013	1.348	−1.356
*P*-value	<0.001	<0.001	<0.001	<0.001	<0.001	0.069	<0.001
**Whole brain**							
ADHD children	1123.41 ± 134.56						
Healthy children	1345.24 ± 125.78						
*T*-value	−3.682						
*P*-value	<0.001						

### ROC analysis results

**FIGURE 4:**
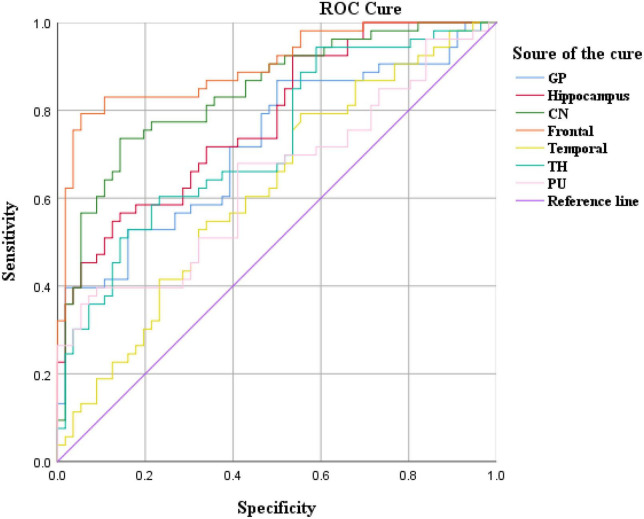
Receiver operating curve (ROC) curve analysis results of CBF in brain regions.

Cerebral blood flow values in brain regions such as the frontal lobe and caudate nucleus distinguished ADHD children (AUC > 0.05, *P* < 0.05), with the highest AUC values in the frontal lobe (Figure 4 and Table 4).

**TABLE 4:** Receiver operating curve (ROC) curve analysis results of CBF in brain regions of ADHD children (*n* = 102).

Brain region	AUC	Std. error	*P*-value	95% CI	
				Lower bound	Upper bound
**CBF**					
Frontal	0.901	0.049	0.000	0.627	0.819
Hippocampus	0.779	0.043	0.061	0.694	0.864
CN	0.841	0.038	0.000	0.767	0.915
PU	0.723	0.030	0.072	0.842	0.959
GP	0.620	0.054	0.084	0.515	0.725
TH	0.728	0.048	0.066	0.634	0.823
Temporal	0.643	0.053	0.091	0.538	0.748

TH, thalamus; GP, globus pallidus; SN, substantia nigra; RN, red nucleus; CN, caudate nucleus; PU, putamen.

## Discussion

The results showed that the frontal lobe volume and CBF values were lower in ADHD children than in healthy children, suggesting that the frontal lobe is less developed in ADHD children; the frontal lobe is located at the forefront of the cerebral hemisphere, occupying the first 1/3 of the surface of the cerebral hemisphere, and is the most advanced part of the developing brain and one of the important neural tissue areas of the body; It includes several functional areas such as cortical motor area, premotor area, cortical lateral visual center, motor speech center, cheirokinesthetic center, frontal association area and urinary and defecation center (28, 29); The functions of the frontal lobes are primarily associated with voluntary movement, and advanced mental functions; for example, the frontal association area, located in the prefrontal lobe, is associated with cognitive, emotional and mental behaviors (30, 31); Frontal lobe is also related to voluntary movement and advanced mental function (32–34). Frontal lobe dysplasia in ADHD children results in abnormal voluntary movement and advanced mental function, and finally leads to hyperactivity and other symptoms.

The results showed that the temporal lobe volume and CBF values were lower in ADHD children than in healthy children, suggesting that the temporal lobe is less developed in ADHD children; the temporal lobe is mainly responsible for processing auditory information, while the temporal lobe also has some relationship with memory and emotion (35). Generally, the temporal lobe is supplied by the middle cerebral artery, which is part of the blood supply of the cerebral anterior circulation (36). Patients with temporal lobe lesions may experience memory loss or emotional disorders, such as mental excitement or depression and emotional disturbance, and in severe cases, hallucinations or delusions may occur, and some patients may also present with temporal lobe epilepsy (37, 38).

The findings showed that the volume and CBF values of brain regions such as caudate nucleus, putamen, and globus pallidus were lower in ADHD children than in healthy children, suggesting abnormal development of basal ganglia brain regions in ADHD children; The basal ganglia are located in the deep brain, consisting of caudate nucleus, lenticular nucleus, putamen, globus pallidus, subthalamic nucleus and substantia nigra (39). The basal ganglia are regulated in concert with the cerebral cortex and cerebellum, and voluntary movement muscular tone and postural reflexes are also involved in the regulation of complex behaviors; lesions in the basal ganglia region mainly result in motion abnormality (increased or decreased movements) and changes in muscle tone (increased or decreased) (40); the basal ganglia region is an important neurological area, closely related to sensory, motor, visual, and behavioral functions (13, 41); in ADHD children, abnormal development of the basal ganglia region may lead to symptoms such as hyperactivity.

The results in Table 2 showed that the volume and CBF values of brain regions such as frontal lobe, temporal lobe, and basal ganglia were lower in ADHD children than in healthy children, suggesting poor development of those brain regions and decreased CBF perfusion in ADHD children. The poor development of frontal lobe, temporal lobe, and basal ganglia in ADHD children may be due to the poor development of brain regions caused by long-term inadequate blood perfusion in these three brain regions, which ultimately leads to the development of clinical symptoms such as hyperactivity in ADHD children (42–44). The results showed that the gray matter volume and CBF values of brain regions such as frontal lobe, temporal lobe and basal ganglia were lower in ADHD children with ADHD than in healthy children, but only the volume of white matter was lower than in healthy children, and the difference in CBF values was not statistically significant, suggesting that the microstructural changes in the brain of ADHD children are mainly in the gray matter, which is probably because there is more blood flow in the gray matter and less blood flow in the white matter. When there is a decrease in blood flow in the gray and white matter at the same time, the same has a more significant impact on the gray matter (45–47).

The results showed that the CBF values of brain regions such as frontal lobe and caudate nucleus could distinguish ADHD children (AUC > 0.5, *P* < 0.05). Therefore, these two brain regions can be used as key brain regions for brain imaging diagnosis of ADHD children.

## Limitations

The study is limited by the fact that it is not a multicenter study and the findings may be somewhat regional in nature; the large age range (5–13 years) of child patients included in the study and the non-inclusion of ADHD children in other age groups may have led to some bias in the findings; the above weaknesses will be further improved in future studies.

## Conclusion

In summary, the 3D-pcASL technique can reflect CBF perfusion and differentiate ADHD children, which may contribute to the diagnosis of ADHD children.

## Data availability statement

The original contributions presented in this study are included in the article/supplementary material, further inquiries can be directed to the corresponding author.

## Ethics statement

This study was approved by the Ethics Committee of the Children’s Hospital of Chongqing Medical University (NO. 2019-221), and the families of children under study signed an informed consent form before the examination. Written informed consent to participate in this study was provided by the participants or their legal guardian/next of kin.

## Author contributions

ST and LH contributed to the experimental design and project management. XL and FQ contributed to the data acquisition and data analysis. WC contributed to the data statistics. LN provided the statistical software support. All authors contributed to the article and approved the submitted version.
