# Corrigendum: Three-dimensional pseudocontinuous arterial spin labeling perfusion imaging shows cerebral blood flow perfusion decline in attention-deficit/hyperactivity disorder children

**DOI:** 10.3389/fpsyt.2023.1156469

**Published:** 2023-02-14

**Authors:** Shilong Tang, Xianfan Liu, Lisha Nie, Fangfang Qian, Wushang Chen, Ling He

**Affiliations:** ^1^Department of Radiology, Children's Hospital of Chongqing Medical University, National Clinical Research Center for Child Health and Disorders, Ministry of Education Key Laboratory of Child Development and Disorders, Chongqing Key Laboratory of Pediatrics, Chongqing, China; ^2^GE Healthcare, MR Research China, Beijing, China

**Keywords:** children, attention-deficit/hyperactivity disorder, magnetic resonance imaging, brain, 3D-pcASL

In the published article, the reference for [35] was incorrectly written as “Jones A, Caes L, Eccleston C, Noel M, Gauntlett-Gilbert J, Jordan A. The sands of time: adolescents' temporal perceptions of peer relationships and autonomy in the context of living with chronic pain. *Paediatr Neonatal Pain*. (2022) 4:110–24. doi: 10.1002/pne2.12071”. It should be “Friederici AD. The brain basis of language processing: from structure to function. *Physiol Rev*. (2011) 91:1357–92. doi: 10.1152/physrev.00006.2011”.

The authors apologize for this error and state that this does not change the scientific conclusions of the article in any way. The original article has been updated.

